# Fusion of a bacterial cysteine desulfurase to redox-sensitive green fluorescent protein produces a highly sensitive cysteine biosensor for monitoring changes in intracellular cysteine

**DOI:** 10.1016/j.redox.2025.103785

**Published:** 2025-07-23

**Authors:** Damien Caubrière, Arthur de Butler, Anna Moseler, Pauline Leverrier, Jean-François Collet, Andreas J. Meyer, Nicolas Rouhier, Jérémy Couturier

**Affiliations:** a Université de Lorraine, INRAE, IAM, F-54000, Nancy, France; b Institute of Crop Science and Resource Conservation (INRES) - Chemical Signalling, University of Bonn, 53117, Bonn, Germany; c WELBIO Department, WEL Research Institute, Avenue Pasteur 6, 1300, Wavre, Belgium; d de Duve Institute, Université Catholique de Louvain, Avenue Hippocrate 75, 1200, Brussels, Belgium; e Institut Universitaire de France, F-75000, Paris, France

**Keywords:** Cysteine desulfurase, Cysteine, Cystine, Fluorescent biosensor, roGFP2

## Abstract

Over the last two decades, the development of fluorescent probes has transformed the way of measuring physiological parameters in intact cells, including in the field of redox biology. We developed a genetically encoded biosensor called CyReB to monitor intracellular cysteine in real time. This biosensor exploits the ability of a particular bacterial cysteine desulfurase to promote the oxidation of reduction-oxidation-sensitive green fluorescent protein 2 in the presence of cysteine. The specificity, sensitivity, and the oxidation-reduction dynamics of CyReB were first investigated *in vitro* before its *in vivo* functionality was confirmed by expressing CyReB in *Escherichia coli* and *Saccharomyces cerevisiae* cells. Expressing CyReB or an inactive version in wild-type and various mutant strains of *Escherichia coli* showed that this sensor could be used to monitor intracellular cysteine dynamics, particularly in the context of the cysteine-cystine shuttle system. This work demonstrates how using this cysteine biosensor should provide new insights into the metabolism of cysteine and cysteine-related pathways in various model organisms.

## Highlights

• CyReB is oxidized by cysteine and reduced by the glutathione-glutaredoxin system.
• CyReB is functional in *Escherichia coli* and *Saccharomyces cerevisiae*.
• GSH and GRXs are important for maintaining a functional cysteine/cystine shuttle.
• CyReB represents a new tool for investigating the cysteine-related pathways.

## Graphical abstract

**Figure:**
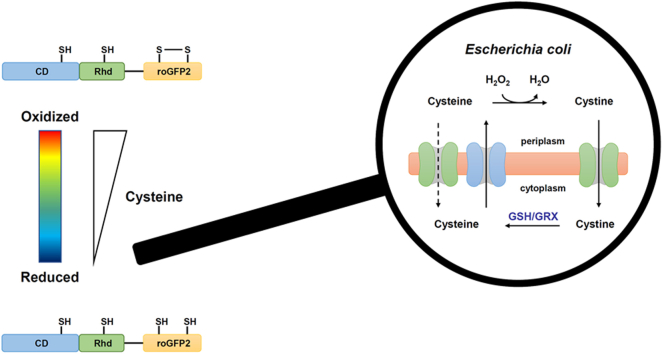

## 1 Introduction

Cysteine is a key metabolite required for the biosynthesis of proteins and many important sulfur-containing molecules. It forms the functional core of glutathione and is involved in methionine biosynthesis in organisms capable of synthesizing it. Cysteine also acts as a source of sulfur for tRNA thio-modification and for important protein cofactors, including biotin, thiamine, lipoic acid, coenzyme A, iron-sulfur cluster, and the molybdenum cofactor [1]. Finally, cysteine is a source of sulfur for hydrogen sulfide (H_2_S), which acts as a gaseous signaling molecule in many physiological processes in most organisms [2]. Due to its toxicity, particularly through its interaction with free iron [3], intracellular cysteine levels are tightly regulated [4,5]. Alterations in cysteine metabolism and variations in H_2_S levels have been reported in many physiological contexts, particularly in cancer, and for bacterial virulence and antibiotic resistance [[4], [5], [6], [7]]. Understanding the role of cysteine in all these processes requires knowledge of how the cellular cysteine pool varies in different physiological situations, cellular compartments, and genetic backgrounds. Due to the presence of cysteine in various subcellular compartments and its susceptibility to oxidation to cystine during extraction, there is great interest in non-invasive methods that can detect cysteine in intact cells with high spatiotemporal resolution.

Genetically encoded biosensors are non-invasive reporters that have been developed to detect and/or quantify a wide array of molecules and physiological parameters in cells [8,9]. Reduction-oxidation-sensitive green fluorescent proteins (namely roGFP1 or roGFP2) have been engineered from GFP and enhanced GFP (eGFP) variants, respectively. These proteins have two cysteine residues on two adjacent β-strands in the protein β-barrel that can form an intramolecular disulfide bond [10,11]. The formation of this disulfide results in a ratiometric change in the chromophore's spectroscopic properties. This change enables a physiological readout that is independent of the biosensor's abundance. In the presence of physiological concentrations of an oxidant, the oxidation of roGFP2 is generally slow, but is accelerated by enzymes that can interact with roGFP2. Coupling roGFP2 to oxidoreductases, such as human glutaredoxin 1, or to yeast Orp1 or Tsa2 thiol peroxidases, results in highly specific probes for detecting changes in the glutathione redox potential (*E*_GSH_) or in hydrogen peroxide (H_2_O_2_) levels, respectively [[12], [13], [14]].

**Fig. 1:**
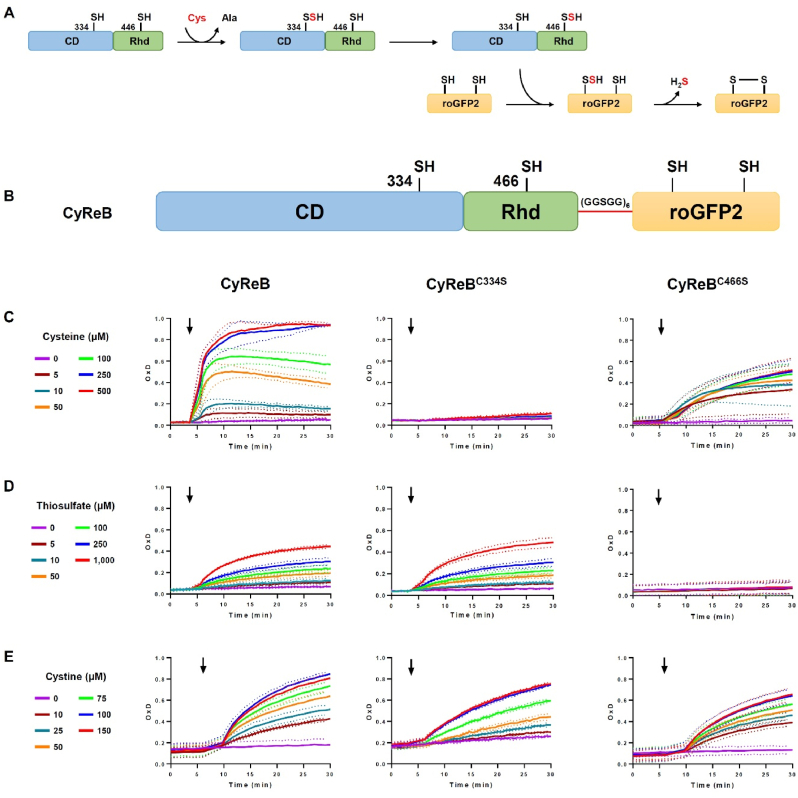
CyReB is efficiently oxidized in the presence of cysteine *in vitro*. (A) Proposed mechanism of cysteine-dependent oxidation of roGFP2 by the bifunctional bacterial CD-Rhd protein that occurs through a series of thiol-persulfide exchange reactions as described in the main text. **(B)** Modular organization of CyReB presenting the fusion of CD-Rhd with roGFP2, which results in a protein with three different modules, i.e., CD (in blue), Rhd (in green), roGFP2 (in orange) separated by a 6xGGSGG linker (in red). The catalytic cysteines of both CD and Rhd domains and cysteines of roGFP2 are also indicated. **(C**–**E)** Degree of oxidation (OxD) of CyReB, CyReB^C334S^ and CyReB^C466S^ (1 μM) in response to increasing concentrations of cysteine (0–500 μM) **(C),** of thiosulfate (0–1000 μM) **(D)** and of cystine (0–150 μM) **(E)**. The arrows indicate the addition of the substrate. The data are represented as mean ± SD of three independent experiments with each three technical replicates.

We have recently demonstrated that a cysteine desulfurase (CD)-rhodanese (Rhd) protein from *Pseudorhodoferax* sp., which consists of fused CD and Rhd domains, was able to catalyze the rapid and complete oxidation of roGFP2 *in vitro* [15]. Cysteine desulfurases are pyridoxal 5′-phosphate (PLP)-dependent homodimers that catalyze the desulfuration of cysteine to alanine while Rhd domain-containing proteins catalyze the transfer of a sulfur atom from sulfur donors to nucleophilic sulfur acceptors. The results showed that the cysteine-dependent oxidation occurred through a series of thiol-persulfide exchange reactions. The intermediate persulfide formed on the reactive catalytic cysteine of the CD domain upon cysteine desulfuration is transferred to the catalytic cysteine of the Rhd domain and then to one of the two cysteines of roGFP2, ultimately leading to the formation of an intramolecular disulfide on roGFP2 and the release of H_2_S (Fig. 1A). In this study, we exploited and combined the biochemical properties of CD-Rhd and roGFP2 to generate a cysteine redox biosensor (CyReB).

## 2 Results and discussion

### 2.1 CyReB is oxidized by cysteine and reduced by the glutathione-glutaredoxin system *in vitro*

CyReB was generated by fusing the CD-Rhd protein from *Pseudorhodoferax* to the N-terminus of roGFP2 via a 30-amino acid linker (GGSGG)_6_ (Fig. 1B). CD-Rhd possesses cysteine desulfurase and thiosulfate sulfurtransferase activities that depend on the catalytic cysteines Cys^334^ and Cys^466^ of the CD and Rhd domains, respectively [15]. Since the four-step reaction of CD-Rhd-mediated roGFP2 oxidation by cysteine involves two thiol-persulfide exchange reactions on the domains involved (Supplementary Fig. 1A) [15], two variants, designated CyReB^C334S^ and CyReB^C446S^, were also produced as recombinant proteins to evaluate the importance of the two catalytic cysteine residues in the CD and Rhd domains of CyReB for roGFP2 oxidation. In the presence of cysteine, pre-reduced CyReB was rapidly oxidized in a concentration-dependent manner, as indicated by the degree of oxidation (OxD) and the initial oxidation rate (Fig. 1C, Supplementary Fig. 1B). CyReB was almost completely oxidized 15 min after the addition of 250 μM cysteine. The requirement of an excess of cysteine for the full oxidation of the probe indicates that the reaction does not reach completion with a stoichiometric concentration of cysteine. This may be because the system reached equilibrium at low concentrations of cysteine or because side-reactions interfere with the persulfide exchange reactions.

Regardless of the cysteine concentration used, CyReB^C334S^ remained mostly reduced throughout all time-course experiments (Fig. 1C). Depending on the concentrations of cysteine added, CyReB^C446S^ was only partially oxidized, reaching OxD values between 30 % and 50 % 25 min after cysteine addition. The initial oxidation rate was independent of cysteine concentration (Fig. 1C, Supplementary Fig. 1C). These results suggest that Cys^334^ in the CD domain is essential for the cysteine-mediated roGFP2 oxidation, and that Cys^446^ in the Rhd domain is necessary for efficient persulfide transfer to roGFP2 and subsequent roGFP2 oxidation (Fig. 1C). The apparent *K*_*M*_ value (35 ± 6 μM) of CyReB for cysteine was lower than the value reported earlier for recombinant CD-Rhd without roGFP2 fusion (273 ± 26 μM) (Supplementary Fig. 1B) [15]. These results indicate that CyReB should be oxidized by cysteine *in vivo*, since the physiological concentrations range from 10 to 300 μM, as previously reported in humans, Arabidopsis, and *E*. *coli* [4,[16], [17], [18]].

**Fig. 2:**
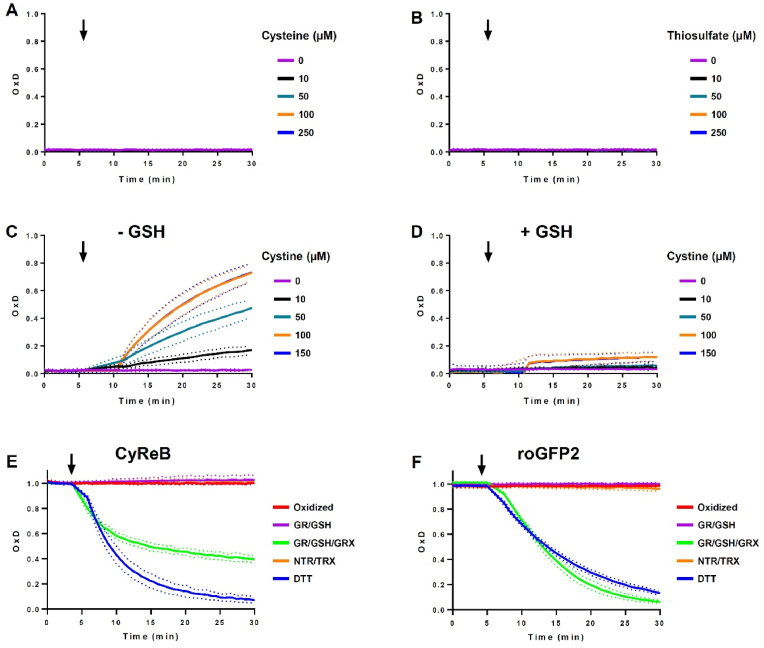
RoGFP2 alone or as part of CyReB is prone to oxidation by cystine in the absence of GSH. Degree of oxidation of roGFP2 (1 μM) in response to cysteine **(A**) or thiosulfate **(B)** at the indicated concentrations. Degree of oxidation of roGFP2 (1 μM) in response to increasing concentrations of cystine (0–150 μM) without **(C)** or with 5 mM GSH **(D)**. The reduction of oxidized (1 μM) CyReB **(E)** or roGFP2 **(F)** was evaluated *in vitro* using either DTT (1 mM), reconstituted GSH/GRX system (200 μM NADPH, 1 U GR, 2 mM GSH and 3 μM poplar GRXC1) or reconstituted TRX system (200 μM NADPH, 200 nM Arabidopsis NTRB and 3 μM poplar TRXh1). The arrow indicates the addition of reductant. Note that the data for NTR/TRX in panels E and F are largely masked by data for the fully oxidized control. The data are represented as mean ± SD of three independent experiments.

Both CyReB and CyReB^C334S^ were slowly and only partially oxidized *in vitro* by 5–1000 μM thiosulfate, the second substrate of CD-Rhd (Fig. 1D, Supplementary Fig. 1D) [15]. In contrast, CyReB^C466S^ was not oxidized at all. These results confirmed that the weak thiosulfate-dependent oxidation of CyReB relies exclusively on the activity of the Rhd domain. This thiosulfate-dependent oxidation of CyReB may likely be neglected as the expected physiological concentration of thiosulfate is assumed to be in the μM range [19]. Finally, we evaluated the sensitivity of CyReB toward cystine, the oxidized form of cysteine, that also represents a physiological source of cysteine for *E. coli* after reduction [5]. All three CyReB versions exhibited a concentration-dependent oxidation reaching 70–80 % oxidation after 30 min (Fig. 1E), suggesting a direct, non-specific oxidation of the roGFP2 module by cystine. To confirm it, we assessed the response of isolated reduced roGFP2 to cysteine, thiosulfate and cystine, respectively (Fig. 2A–C). While roGFP2 was insensitive to cysteine and thiosulfate, it was gradually oxidized by cystine in a concentration-dependent manner, very similar to the oxidation observed for CyReB. However, this effect was no longer visible in the presence of 5 mM GSH (Fig. 2D), and the intracellular cystine concentration should be very low at least in reducing compartments. Based on these results, we concluded that neither thiosulfate nor cystine should significantly affect the oxidation state of CyReB in physiological conditions.

The cellular redox state of roGFP2-based sensors is determined by the relative rates of oxidation through the sensing module and of reduction, which depends on the GRX/GSH system [11,20]. We confirmed that cysteine-oxidized CyReB is reduced by GSH *in vitro* in the presence of GRX, but not by the NADPH thioredoxin reductase/thioredoxin (NTR/TRX) system (Fig. 2E). Noticeably, the reduction of CyReB by NADPH/GR/GSH in the presence of GRX was less efficient than the reduction of free roGFP2 (Fig. 2F). This difference may reflect the fact that the CD-Rhd module competes with GRXC1 for access to the thiols of the roGFP2 module. Here, it is important to note that the apparent concentration of the fused CD-Rhd in the vicinity of the roGFP2 thiols is likely higher than the apparent concentration of non-fused GRXC1, which is present at 3 μM. Overall, the specificity, sensitivity and reversibility of the cysteine-dependent oxidation of CyReB pave the way for real-time *in vivo* measurements. However, given that CyReB consumes cysteine to oxidize roGFP2, it will not provide information about the absolute intracellular cysteine concentration. In summary, the new biosensor should allow monitoring dynamic variations in the intracellular cysteine levels in subcellular compartments where GSH and GRX are present, i.e., the cytosol and other organelles after specific targeting. Using CyReB^C334S^ as a control should ensure that the variations detected are indeed specific to cysteine.

### 2.2 CyReB is functional in *E. coli* and *Saccharomyces cerevisiae*

**Fig. 3:**
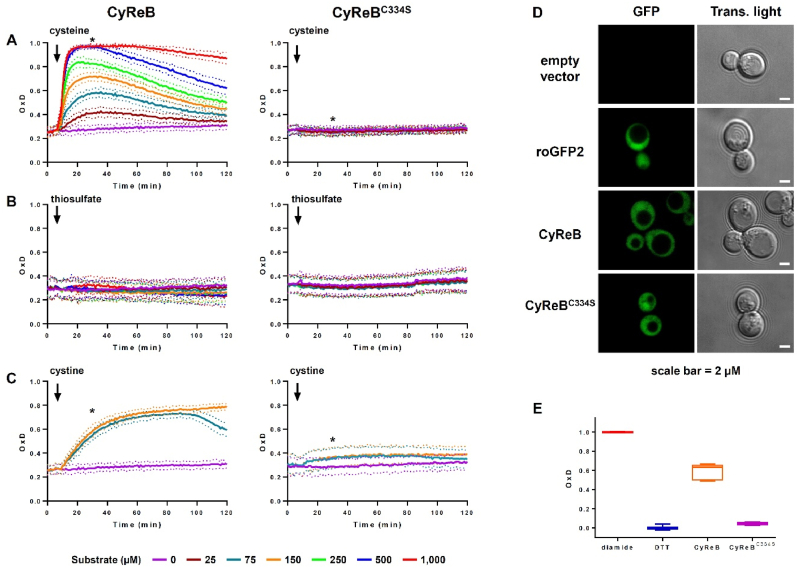
CyReB is a specific cysteine sensor, functional in *E. coli* and *S. cerevisiae*. Degree of oxidation of CyReB and CyReB^C334S^ expressed in *E. coli* BL21(DE3) cells, to the addition of exogenous cysteine **(A)**, thiosulfate **(B)** or cystine **(C)** at the indicated concentrations. The arrows indicate the addition of the substrate, and the stars indicate the 30 min time point used in Fig. 4. Each experiment was performed with cells obtained from independent transformation events. For each probe, the data are represented as mean ± SD of three independent experiments in which the response to each substrate concentration was measured with cells obtained from three independent colonies (*n* = 9 biological replicates). **(D)** Representative confocal microscopy images of BY4742 yeast cells transformed with the empty vector or expressing roGFP2, CyReB or CyReB^C334S^. Shown is the GFP fluorescence collected at 505–530 nm after excitation at 488 nm. Scale bar = 2 μm. **(E)** Fluorescence measurement of CyReB and CyReB^C344S^ under steady-state conditions. Values were calculated relative to those obtained for fully oxidized (diamide) and fully reduced (DTT) CyReB and CyReB^C344S^, respectively. The box plot shows the median as center line with the box for the first to the third quartile and whiskers indicating min and max values of the whole data set.

The *in vivo* functionality of CyReB was first investigated in *E. coli* BL21(DE3) by monitoring the oxidation levels of both CyReB and CyReB^C334S^ before and after the addition of cysteine, thiosulfate, or cystine (Fig. 3A–C). Cysteine can be imported non-specifically by branched-chain amino acid transporters (LIV system) [21] or specifically by the transporter CyuP when cysteine is abundant [22]. In *E. coli*, the *CyuP* gene is part of an operon that serves to detoxify cysteine, notably under anoxic conditions, together with *CyuA* [22]. CyuA is a cysteine desulfidase/desulfhydrase that breaks down cysteine into pyruvate, ammonia and H_2_S. Other cysteine consuming pathways are dependent on the cysteine desulfidase activity of the tryptophanase TnaA [23] or on the combined action of the amino transferase AspC and of the 3-mercaptopyruvate sulfurtransferase MstA. The first enzyme converts cysteine into 3-mercaptopyruvate, which the second enzyme then degrades into pyruvate and H_2_S [24]. Thiosulfate is efficiently imported by a sulfate/thiosulfate permease [25], serving for the synthesis of S-sulfocysteine, which is then converted to cysteine [26]. Cystine is imported by two high-affinity importers, TcyJLN and TcyP, with apparent *K*_*M*_ values of 30 nM and 2 μM, respectively, and is in principle rapidly reduced to cysteine by the GSH/GRX system [27].

At steady state, *i.e.,* before the addition of the substrate, both CyReB and CyReB^C334S^ were slightly oxidized, with an OxD of ∼0.25–0.30. This level of oxidation remained stable for 2 h. The addition of cysteine rapidly oxidized CyReB but not CyReB^C334S^. The initial oxidation rate and the maximum OxD were proportional to the cysteine concentration added (Fig. 3A, Supplementary Fig. 1E–F). Detectable oxidation of CyReB occurred after the addition of 25 μM cysteine, and full oxidation was reached with the addition of 500 μM cysteine. In all cases, OxD peaked at about 15–30 min after cysteine addition and subsequently declined gradually over the following 90 min. Only after the addition of 1 mM cysteine was the maximum oxidation maintained for approximately 60 min before the OxD again began to decline gradually (Fig. 3A). A progressive reduction of CyReB was visible at all concentrations a few minutes after cysteine addition (Fig. 3A). Although CyReB was never returned to the initial steady-state level after these 2-h experiments, we demonstrated that this oxidation is fully reversible with the addition of DTT (Supplementary Fig. 1G).

Neither CyReB nor CyReB^C334S^ were oxidized when thiosulfate was added to the cells (Fig. 3B). This indicates that the amount of internalized thiosulfate was insufficient to oxidize CyReB via the Rhd domain and/or that the addition of thiosulfate did not promote sufficient cysteine synthesis in the *E. coli* cytosol under these experimental conditions. Adding cystine resulted in a slow and progressive CyReB oxidation, with an increase in OxD for about 30 min before reaching a plateau. The duration of the plateau depended on the concentration used (Fig. 3C). Since CyReB^C334S^ was not significantly oxidized (Fig. 3C), we concluded that CyReB oxidation reflects the increase in cysteine levels upon cystine reduction [16]. The initial oxidation rate of CyReB induced by cystine is slower than the oxidation rate observed with the addition of equivalent amounts of cysteine (i.e., 75 μM cystine versus 150 μM cysteine). Since the cystine concentrations used were well above the affinities of both cystine importers (30 nM and 2 μM), the importers should be saturated. Thus, the oxidation of CyReB is very likely limited by cystine uptake, rather than by cystine reduction with an estimated intracellular reaction rate of 70 mM/min [27].

To validate CyReB as a functional cysteine biosensor in eukaryotes, CyReB and CyReB^C334S^ were also expressed in *Saccharomyces cerevisiae*. Both constructs, as well as roGFP2, were expressed in the cytosol without affecting cell growth (Fig. 3D, Supplementary Fig. 2A). At steady state, CyReB was partially oxidized (OxD of ∼0.5/0.6), while CyReB^C334S^ was mostly reduced (Fig. 3E). The oxidation of CyReB indicates that the probe can sense cytosolic cysteine in yeast cells. The addition of exogenous cysteine, but not thiosulfate, sulfate, N-acetyl-cysteine, or sodium sulfide, promoted the further oxidation of CyReB (Supplementary Fig. 2B–F). Cystine was not tested due to the absence of a cystine transporter in *S. cerevisiae* [28]. Taken together, these results suggest that CyReB functions both in *E. coli* and *S. cerevisiae*, specifically sensing changes in cysteine levels.

### 2.3 Investigating the role of cellular reductants for the cysteine/cystine shuttle system in *E. coli* using CyReB

In *E. coli*, the cytoplasmic cysteine concentration (approximately 55 μM) [16] is highly regulated and the cysteine/cystine shuttle system is an important mechanism for maintaining cysteine homeostasis [5,16]. To avoid cysteine toxicity [14,25], a significant amount of cysteine is specifically exported to the periplasm and oxidized to cystine, even in the absence of oxidative stress [27,29]. At some point, cystine can then be imported back into the cytoplasm and reduced to cysteine [16,27,30]. Therefore, we sought to investigate the role of the major reducing systems in this cysteine/cystine shuttle system by expressing CyReB and CyReB^C334S^ in three mutant strains, Δ*gshA*, Δ*grxA/B/C* and Δ*trxA/C*. The Δ*gshA* variant is defective for γ glutamate-cysteine ligase, which catalyzes the first step of glutathione synthesis. The Δ*grxA/B/C* mutant lacks all redox-active Grxs, and the Δ*trxA/C* mutant lacks both *trxA* and *trxC* genes, so it no longer possesses conventional Trx proteins.

**Fig. 4:**
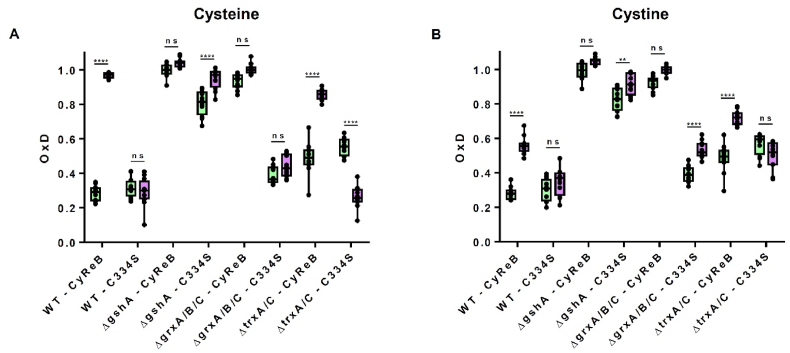
A functional GSH/glutaredoxin system is required for efficient cystine reduction in *E. coli*. Degrees of oxidation of CyReB and CyReB^C334S^ expressed in *E. coli* BL21(DE3) WT, Δ*gshA*, Δ*grxA/B/C* or Δ*trxA/C* cells were determined before (green) and 30 min after the addition of either exogenous cysteine (500 μM) **(A)** or cystine (75 μM) (violet) **(B)**. The box plots are derived from the datasets presented in Supplementary Figs. 3 and 4. The box plots show the median as center line with the box for the first to the third quartile and whiskers indicating min and max values of the whole data set. Values (circles) correspond to two technical repeats of six independent biological replicates. Significance test was performed using two-way ANOVA with Tukey's multiple comparison post-hoc test. ns > 0.05, ∗∗p < 0.01, ∗∗∗∗p < 0.0001 (Supplementary Tables 1 and 2).

At steady state, CyReB was almost completely oxidized in both Δ*gshA* and Δ*grxA/B/C* cells, but only half oxidized in Δ*trxA/C* cells (Supplementary Figs. 3, 4, Fig. 4). While the steady-state oxidation of CyReB^C334S^ and CyReB was similar in WT and Δ*trxA/C* cells, CyReB^C334S^ was reduced more than CyReB in Δ*gshA* cells and even more in Δ*grxA/B/C* cells. The difference in oxidation of CyReB compared to CyReB^C334S^ reflects the cysteine-specific oxidation of CyReB that occurs in the cytoplasm and the lack of reduction upon oxidation. Since this difference was observed only in Δ*gshA* and in Δ*grxA/B/C* cells, these results clearly show that a functional GSH/GRX system is required for CyReB reduction *in vivo*, which is consistent with the *in vitro* observation (Fig. 2E), while the TRX system is not. The increased oxidation of both CyReB^C334S^ and CyReB in the Δ*trxA/C* background compared to WT cells (OxD ∼0.6 vs. ∼0.25, Fig. 3C vs. Fig. 3A) may be due to a generally less reducing environment and in particular an indirect effect on the concentration of reduced versus oxidized form of glutathione.

The high steady-state oxidation of CyReB in both Δ*grxA/B/C* and Δ*gshA* cells prevents adequate dynamic monitoring of the intracellular cysteine concentration in response to exogenous supplementation of cysteine or cystine. Accordingly, a shift in CyReB oxidation upon the addition of cysteine or cystine to these mutant strains is visible but not considered statistically significant (Fig. 4, Supplementary Figs. 3 and 4). In Δ*trxA/C* cells, the oxidation pattern of both CyReB and CyReB^C334S^, in response to exogenous addition of cysteine or cystine, was comparable to that of WT cells with a specific oxidation of CyReB (Fig. 4, Supplementary Figs. 3C and 4C). Note that for clarity, the data shown in Fig. 4 only represent OxD values determined before and 30 min after the addition of 500 μM cysteine or 75 μM cystine, respectively (Fig. 4A and B). However, the full response curves are available (Supplementary Figs. 3 and 4). This corresponds to the time point where the OxD value of CyReB was at its maximum following the addition of cysteine to *E. coli* cells, regardless of the concentration used (Fig. 3A).

The absence of oxidation of CyReB^C334S^ in Δ*trxA/C* cells after addition of cystine, in contrast to both Δ*gshA* and Δ*grxA/B/C* (Fig. 4, Supplementary Fig. 4), demonstrates that the TRX system is not involved in cystine reduction in *E. coli*, in contrast to GSH and GRX as reported previously [27]. This is in opposition to the situation observed in humans, mice and *Caenorhabditis elegans*, in which the TRX-related protein, TRP14, acts as a cytosolic cystine reductase [31]. Moreover, the response of CyReB^C334S^ to both cysteine and cystine provides additional evidence for the involvement of GSH and/or GRX in the cysteine/cystine shuttle system. Indeed, the redox state of CyReB^C334S^ reached full oxidation in response to increasing cysteine concentrations in Δ*gshA* cells, whereas the OxD remained at values of approximately 0.4 in Δ*grxA/B/C* cells and was even further decreased in Δ*trxA/C* cells with increasing cysteine concentrations (Fig. 4A, Supplementary Fig. 3). Thus, providing an excess of cysteine to bacterial cells unable to synthesize GSH promoted non-specific oxidation of the roGFP2 domain of CyReB. This oxidation is likely due to the progressive oxidation of exported cysteine in the periplasm, forming cystine that is reimported, although it has been reported that an excess of cysteine is less excreted and cystine import is slower in Δ*gshA* cells compared to WT cells [27]. The fact that CyReB^C334S^ was more oxidized in Δ*gshA* cells suggests that the imported cystine, whether exogenous or resulting from cysteine excretion into the periplasm and subsequent oxidation, was not efficiently reduced in the absence of GSH. This appears to be consistent with the observation that 5 mM GSH prevented the *in vitro* oxidation of roGFP2 by cystine (Fig. 2D). Notably, in contrast to Δ*gshA* cells, cystine import is unaffected in Δ*grxA/B/C* cells and cystine addition triggers the overexpression of the TcyP importer and increased cystine import [27]. Therefore, the oxidation of CyReB^C334S^ observed in Δ*grxA/B/C* cells in the presence of cystine (but not of cysteine) could be the consequence of cystine accumulation that cannot be fully reduced by GSH alone (Fig. 4). This confirmed that GRXs catalyze the GSH-dependent reduction of cystine and that both GSH and GRXs are important for cystine reduction and for maintaining a fully functional cysteine/cystine shuttle system.

In conclusion, we have provided evidence that the genetically encoded biosensor, CyReB, is a functional non-invasive reporter for assessing dynamic changes of cysteine levels in both *E. coli* and *S. cerevisiae*. This novel tool is expected to pave the way for new and fundamental insights into cysteine metabolism and cysteine-related pathways.

## 3 Methods

### 3.1 Reagents

Ammonium sulfate, d-cysteine, diamide, glutathione, glutathione reductase (GR) from baker's yeast, H_2_O_2_, l-cysteine, N-acetyl-l-cysteine, pyridoxine hydrochloride and sodium thiosulfate were purchased from Sigma-Aldrich. l-cystine and sodium sulfide (Na_2_S) nonahydrate were purchased from Acros Organics. Dithiothreitol (DTT), ethylenediaminetetraacetic acid (EDTA), imidazole, isopropyl-β-d-thiogalactopyranoside (IPTG) and phenylmethylsulfonyl fluoride (PMSF) were purchased from Euromedex.

### 3.2 Expression constructs

The CyReB construct consists of a fusion between the sequence encoding the CD-Rhd fusion protein from *Pseudorhodoferax* sp. Leaf274 [15], a 6xGGSGG linker repeat and the sequence encoding roGFP2 with a C-terminal His_6_ tag. For recombinant protein expression in Rosetta2(DE3), synthetic sequences (Genecust) coding for CyReB, CyReB^C334S^ and CyReB^C466S^ were cloned into the *Nco*I and *Xho*I restriction sites of pET-28a. For expression in BL21(DE3) *E*. *coli* strain, synthetic codon-optimized sequences (Genecust) of CyReB and CyReB^C334S^ cloned into the *Nco*I and *Xho*I restriction sites of pET15b were used. For expression in yeast, synthetic codon-optimized sequences of CyReB and CyReB^C334S^ (Genecust) were cloned via the Gateway system into pAG415GPD-ccdB (Addgene Plasmid #14146).

### 3.3 Expression in *E. coli* and purification of recombinant proteins

CyReB, CyReB^C334S^ and CyReB^C466S^ were expressed in *E. coli* Rosetta2(DE3) pLysS strain and purified as described previously [15]. The recombinant proteins were concentrated by ultrafiltration, dialyzed and finally stored in 50 mM Tris-HCl pH 8, 300 mM NaCl supplemented with 10 mM DTT and 50 % glycerol at −20 °C. Protein concentrations were determined spectrophotometrically using a molar extinction coefficient at 280 nm of 69,330 M^−1^ cm^−1^ for CyReB, CyReB^C334S^ and CyReB^C466S^. The *Arabidopsis thaliana* NTRB and poplar GRXC1 and TRXh1, and roGFP2 recombinant proteins, have been purified as described [11,[32], [33], [34]].

### 3.4 Reduction and oxidation of purified recombinant proteins

Prior to reduction, glycerol was removed by ultrafiltration and dialysis at 4 °C using a Vivaspin 6 centrifugal concentrator (10,000 MWCO, Sartorius). The purified proteins were then reduced with freshly prepared DTT (10 mM) for 1 h at room temperature. Excess DTT was removed using desalting spin columns (Zeba spin 7k MWCO, Thermo Fischer Scientific) pre-equilibrated with 30 mM Tris-HCl pH 8, 200 mM NaCl. Fully oxidized CyReB was obtained by incubating reduced protein with 10 mM H_2_O_2_ for 1 h and desalted as described above.

### 3.5 Measurement of the *in vitro* oxidation/reduction of CyReB

Ratiometric fluorescence measurements were performed in 30 mM Tris-HCl pH 8, 1 mM EDTA, at 25 °C, using the EnSight multimode plate reader (PerkinElmer), with excitation at 400 ± 10 and 480 ± 10 nm and fluorescence detection at 520 nm with a bandwidth of 10 nm. Time-course experiments were performed with 1 μM reduced CyReB, CyReB^C334S^ and CyReB^C466S^, or 1 μM oxidized CyReB, in a final volume of 100 μL per well of a 96-well plate (Optiplate 96 F, black, PerkinElmer). Fluorescence intensities for both excitation channels were recorded for 3–5 min before the addition of substrates, and the measurements were continued for another 30 min. CyReB oxidation was performed by addition of cysteine (0–500 μM), thiosulfate (0–1 mM) or cystine (0–150 μM) alone or of cystine with 5 mM GSH. CyReB reduction was followed after addition of 1 mM DTT, of the GRX/GSH system (200 μM NADPH, 0.5 U GR, 2 mM GSH and 3 μM GRXC1) or of the TRX system (200 μM NADPH, 200 nM NTRB and 3 μM TRXh1). In these cases, NADPH, GR and GSH or NADPH and NTR were added initially to the reaction mixture with oxidized CyReB. The reduction reaction was initiated by adding GRX or TRX. Controls for fully reduced or fully oxidized probes were obtained after preincubation with 10 mM DTT or H_2_O_2_ for 1 h, respectively.

### 3.6 Generation of *E. coli* strains

*E. coli* BL21(DE3) was used as wild type throughout the study. Δ*gshA*, Δ*grxA/B/C* and Δ*trxA/C* mutant strains were generated by P1 phage transduction. The corresponding alleles, from the KEIO (kan^R^) collection [35], were transferred into BL21(DE3) wild type background. Insertion of kanR resistance cassette was assessed by PCR. To generate multiple deletions (Δ*grxA/B/C* and Δ*trxA/C*), single mutants (*grxA* and *trxA*) or double mutant (*grxA/B*) were transformed by pCP20 plasmid to excise the resistance cassette, as described [36], before being transduced again using P1 phage.

### 3.7 Measurement of CyReB and CyReB^C334S^ redox state in *E. coli* cells

All experiments were performed with freshly transformed *E. coli* BL21(DE3) cells (pET15b plasmids) and grown in LB medium supplemented with 50 μg/mL ampicillin for BL21(DE3) strain or with 50 μg/mL ampicillin and kanamycin for BL21(DE3) mutant strains (Δ*gshA*, Δ*grxA/B/C* and Δ*trxA/C*). A single colony was used to inoculate a 200 mL LB culture at 37 °C. The next day, a 400 mL LB culture was inoculated at an OD_600nm_ of 0.05 from the overnight culture and supplemented with the appropriate antibiotics and 100 μM pyridoxine hydrochloride. After growing the cells at 37 °C to an OD_600nm_ of 0.3, the expression of CyReB and CyReB^C334S^ was induced by the addition of 100 μM IPTG for 2 h at 20 °C. After reaching an OD_600nm_ of 0.6, bacterial cells were harvested by centrifugation at 6380 *g* for 10 min at 25 °C, and washed with 100 mM phosphate buffered saline (PBS) pH 7.0, 1 mM EDTA. After a second centrifugation at 6380 *g* for 10 min at 25 °C, bacterial cells were resuspended in 100 mM PBS pH 7.0, 1 mM EDTA to reach an OD_600nm_ of 4. The cell suspensions were incubated at 37 °C for 15 min before transferring 190 μL per well of a 96-well plate (Optiplate 96 F, black, PerkinElmer). Fluorescence intensities for both excitation wavelengths were measured for 6.4 min before and for 120 min after the addition of 10 μL of concentrated solutions of the different compounds tested (cysteine, thiosulfate, and cystine). *E. coli* cells expressing an empty pET15b vector were used as controls to subtract background autofluorescence. Fully reduced and oxidized conditions were generated from treatments with 10 mM DTT and 5 mM diamide, respectively.

### 3.8 Measurement of the CyReB redox state in *Saccharomyces cerevisiae* cells

The yeast BY4742 strain was transformed as described previously [37]. For analyzing expression and subcellular localization, colonies were inoculated overnight in selection medium lacking leucine and analyzed for fluorescence the next day. Yeast cells were imaged using a Zeiss LSM 780 confocal laser scanning microscope (CLSM) connected to an Axio Observer. Z1 (Carl Zeiss Microscopy, Jena, Germany) with a 63x lens (Plan‐Apochromat 63x/1.4 Oil DIC M27). RoGFP2 fluorescence was measured by excitation at 488 nm with emission recorded at 505–530 nm. RoGFP2 assays were performed as described previously using a BMG Labtech CLARIOstar fluorescence plate reader [38,39]. Growth rates were measured in a volume of 280 μL at 28 °C using a plate reader (POLARstar Omega) in 96-well plates (Thermo Scientific, NUNC 96-Well) monitoring the increase in absorbance at 600 nm starting from an initial OD_600_ of 0.2.

### 3.9 Calculation of the degree of oxidation of roGFP2

The degree of oxidation (OxD) was calculated as described previously [40] according to the following equation:

$$\text{OxD}\;\text{roGFP}2=\frac{R-Rred}{\left(I480ox/I480red\right)\left(Rox-R\right)+\left(R-Rred\right)}$$

R is the ratio of fluorescence intensities measured at 520 nm after excitation at 400 and 480 nm, respectivly. R_red_ and R_ox_ represent the fluorescence ratios of fully reduced and fully oxidized roGFP2, respectively. The raw intensity values (I) were always corrected for the blank values obtained with buffer alone.

## CRediT authorship contribution statement

**Damien Caubrière:** Writing – original draft, Investigation, Data curation. **Arthur de Butler:** Investigation, Data curation. **Anna Moseler:** Writing – review & editing, Investigation, Data curation. **Pauline Leverrier:** Writing – review & editing, Methodology. **Jean-François Collet:** Writing – review & editing, Methodology. **Andreas J. Meyer:** Writing – review & editing, Formal analysis. **Nicolas Rouhier:** Writing – review & editing, Supervision, Formal analysis. **Jérémy Couturier:** Writing – review & editing, Writing – original draft, Supervision, Funding acquisition, Formal analysis, Conceptualization.

## Funding sources

This work was supported by the 10.13039/501100001665Agence Nationale de la Recherche as part of the ‘Investissements d’Avenir’ program (ANR-11-LABX-0002-01, Lab of Excellence 10.13039/100015818ARBRE), and by « Lorraine Université d’Excellence», as part of the France 2030 Program (ANR-15-IDEX-04-LUE). A.M. was supported by a fellowship from the 10.13039/100005156Humboldt Foundation and is grateful to the 10.13039/501100008131University of Bonn for additional funding.

## Declaration of competing interest

The authors declare no competing interest.

## Data Availability

Data will be made available on request.
