# Supplementary material for: Fusion of a bacterial cysteine desulfurase to redox-sensitive green fluorescent protein produces a highly sensitive cysteine biosensor for monitoring changes in intracellular cysteine
Source: Redox Biol. 2025 Jul 23;85:103785. doi: 10.1016/j.redox.2025.103785 (PMC12319549; doi:10.1016/j.redox.2025.103785)
Supplement: Multimedia component 1 [file mmc1.docx]

**Appendix A. Supplementary Figures**


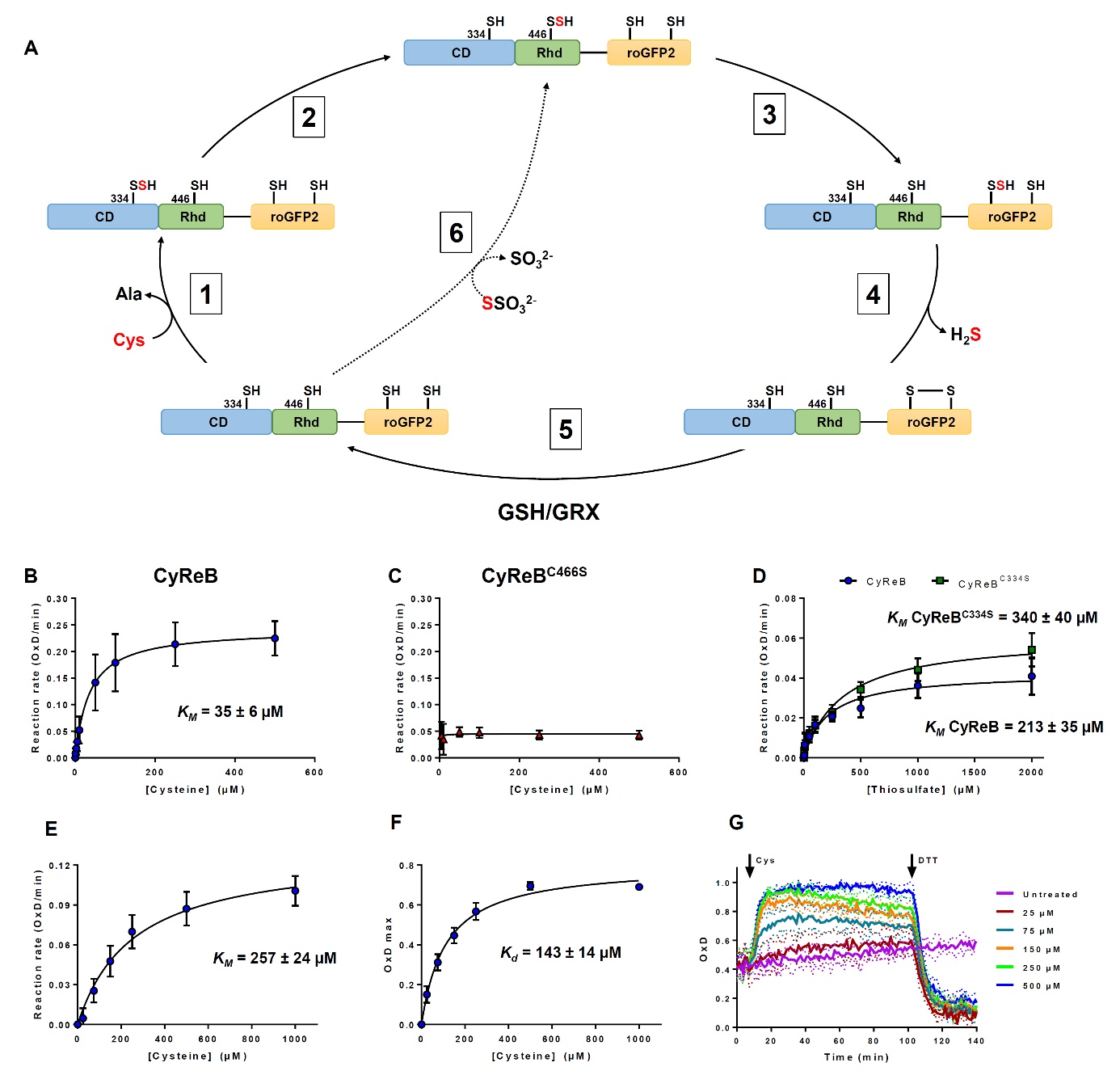


**Supplementary Fig. 1. *In vitro* and *in vivo* characterization of CyReB. (A)** Proposed mechanism of cysteine- or thiosulfate-dependent oxidation of CyReB. The catalytic cysteine (Cys^334^) of the CD domain (in blue) catalyzes the cysteine desulfuration, leading to its persulfidation and the concomitant release of alanine (1). Then, the sulfur atom is transferred to the catalytic cysteine (Cys^466^) of the Rhd domain (in green) through a transpersulfidation reaction (2). Another transpersulfidation reaction allows sulfur transfer to one of the two cysteine residues of roGFP2 (in orange) (3) prior to the formation of the intramolecular disulfide bridge and the concomitant release of hydrogen sulfide (H_2_S) (4). The GSH/GRX system catalyzes the reduction of roGFP2 disulfide bridge (5). Cys^466^ is also responsible for thiosulfate sulfurtransferase activity through the conversion of thiosulfate into sulfite (6). This side reaction was observed *in vitro* using non-physiological thiosulfate concentrations. The *in vitro* reaction rate of CyReB **(B)**, of CyReB^C466S^ **(C)** for cysteine, and CyReB or CyReB^C334S^ **(D)** for thiosulfate, were calculated as linear regression for the first 3 min after addition of substrate from the datasets presented in Fig. 1B and Fig. 1D, respectively. All these data are represented as mean ± SD of three technical replicates of three independent experiments. The degree of oxidation of CyReB expressed in *E. coli* BL21(DE3) cells is proportional to the concentration of cysteine exogenously applied. **(E)** The reaction rate of CyReB in *E. coli* BL21(DE3) cells was calculated as linear regression for the first 5 min after exogenous addition of cysteine, derived from the datasets presented in Fig. 3A. **(F)** The evolution of maximal OxD values measured for each exogenous cysteine concentration applied was represented using one binding total model: OxD = OxDmax*[Cysteine]/(Kd+[Cysteine]) + NS*[Cysteine] + OxD without cysteine. NS corresponds to the slope of the nonlinear regression and is expressed as OxD units divided by [Cysteine] units. **(G)** The oxidation of CyReB expressed in *E. coli* BL21(DE3) cells is reversible. Degree of oxidation of CyReB was monitored following the addition of exogenous cysteine at the indicated concentrations and subsequent addition of 1 mM DTT. Arrows indicate the time point of cysteine or DTT addition. The data are represented as mean ± SD of three independent biological replicates.

**
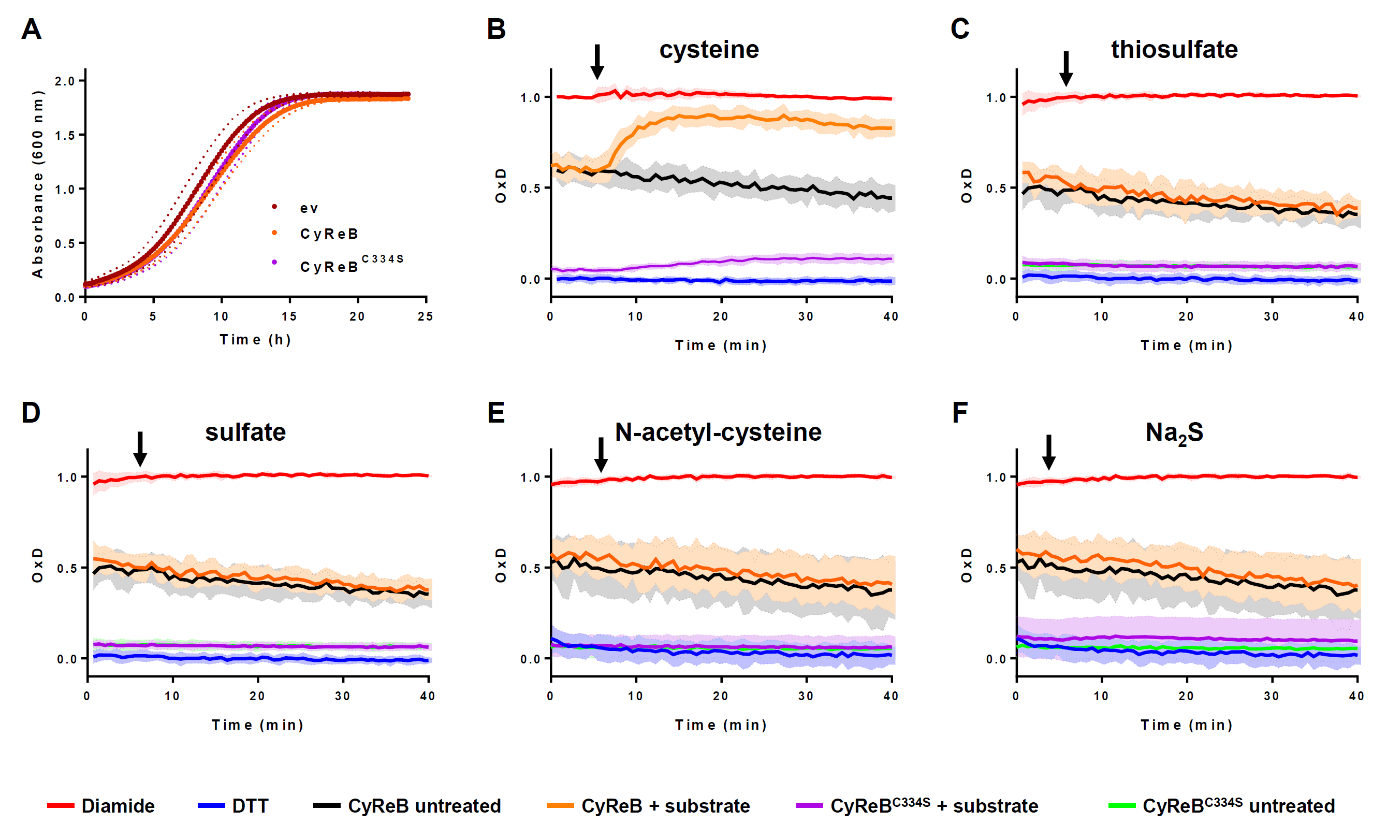
**

**Supplementary Fig. 2. Cysteine specifically promotes oxidation of CyReB in *S. cerevisiae*.**

**(A)** Growth rate of yeast cells expressing CyReB or CyReB^C334S^ in the cytosol. Growth rates were measured at 28°C in a volume of 280 µL in 96-well plates (Thermo Scientific, NUNC 96-Well) using a plate reader (POLARstar Omega) by monitoring the increase in absorbance at 600 nm starting from an initial OD_600_ of 0.2. **(B-F)** Change in degree of oxidation of CyReB and CyReB^C334S^ expressed in yeast cells upon addition of 250 µM cysteine **(B)**, 250 µM thiosulfate **(C)**, 250 µM sulfate **(D)**, 250 µM N-acetyl-L-cysteine **(E)** or 250 µM Na_2_S **(F)**. Experiments in (C) and (D) as well as in (E) and (F) were performed side by side and share the same controls. Arrows indicate the time point of substrate addition. The data are represented as mean ± SD of three biological replicates.


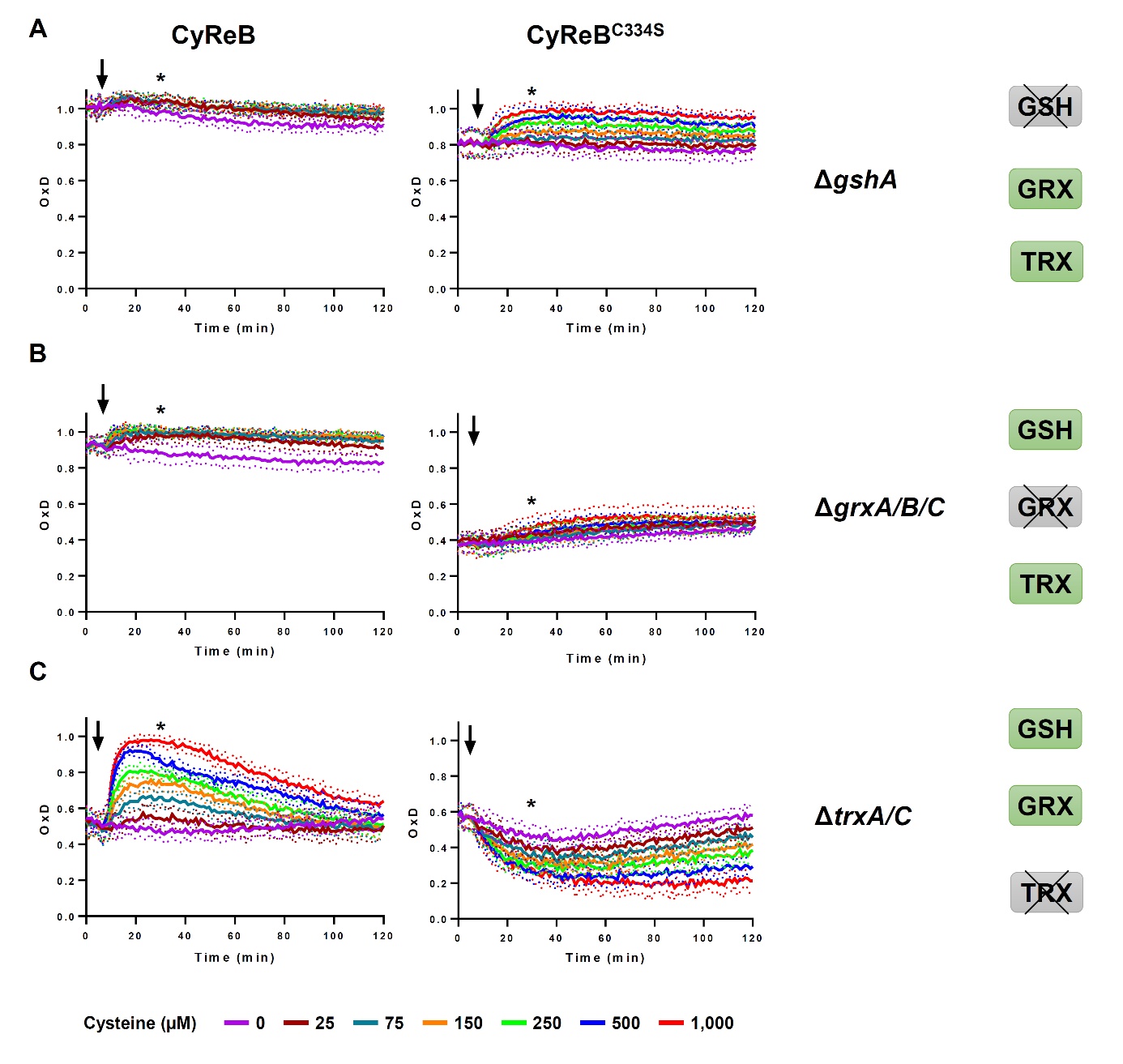


**Supplementary Fig. 3. Excess of cysteine promotes the oxidation of CyReB^C334S^ when GSH synthesis is not functional.** Degree of oxidation of CyReB and CyReB^C334S^ expressed in *E. coli* BL21(DE3) Δ*gshA* **(A)**, Δ*grxA/B/C* **(B)** or Δ*trxA/C* cells **(C)**, in response to exogenous cysteine concentrations ranging from 0 to 1,000 µM. Each experiment was performed with cells obtained from independent transformation events. The data are represented as mean ± SD of two independent experiments in which response to each substrate concentration was measured with cells obtained from three independent colonies (*n* = 6 biological replicates). The arrows indicate the addition of the substrate, and the stars indicate the 30 min time points, which were subsequently used in the analysis presented in Figure 4.


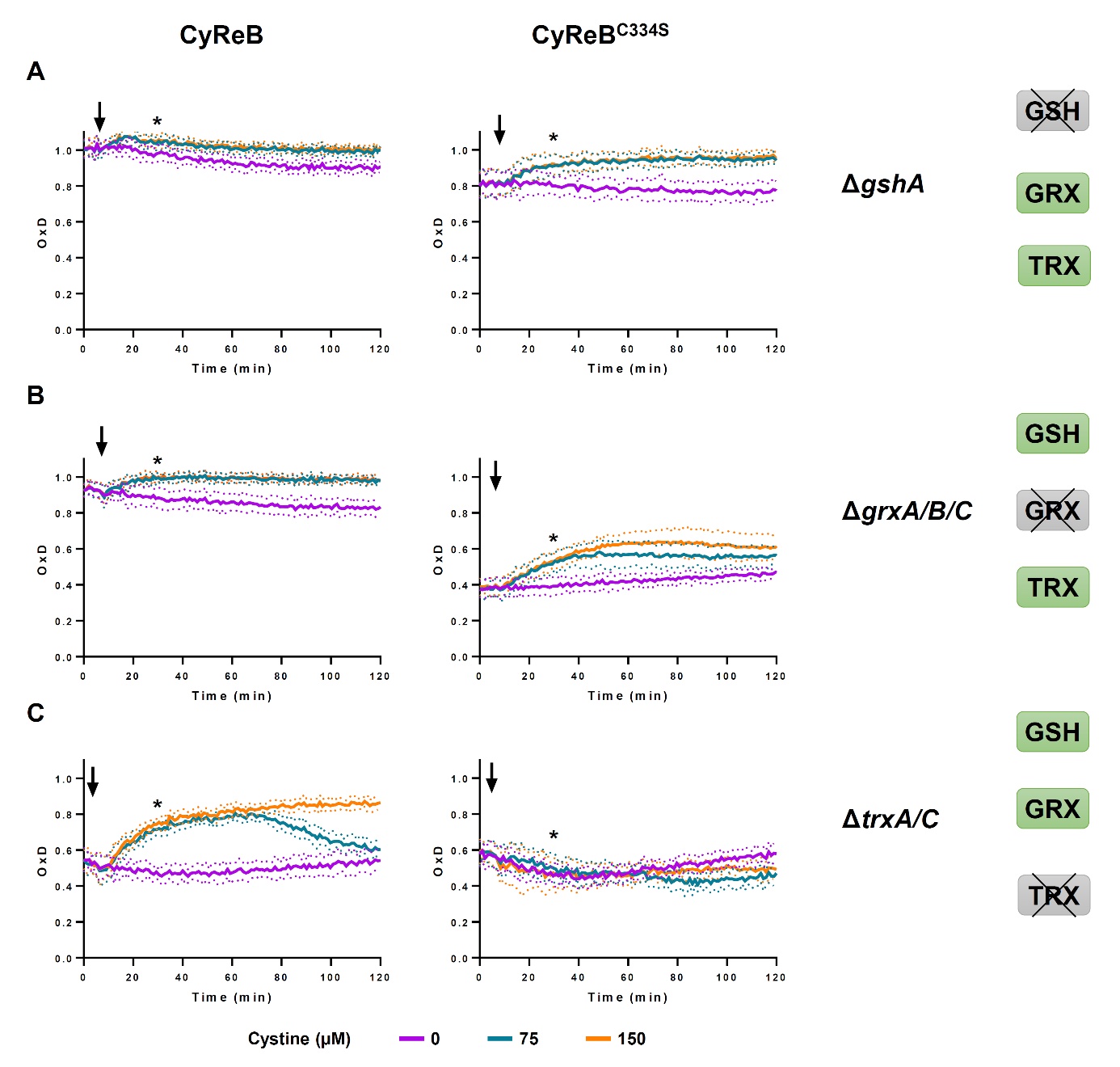


**Supplementary Fig. 4. Cystine reduction relies on GSH/GRX system in *E. coli*.**

Response of CyReB and CyReB^C334S^ expressed in *E. coli* BL21(DE3) Δ*gshA* **(A)**, Δ*grxA/B/C* **(B)** or Δ*trxA/C* cells **(C)**, to exogenous cystine concentrations ranging from 0 to 150 µM. Each experiment was performed with cells obtained from independent transformation events. The data are represented as mean ± SD of two independent experiments in which response to each substrate concentration was measured with cells obtained from three independent colonies (*n* = 6 biological replicates). The arrows indicate the addition of the substrate and the stars indicate the 30 min time points, which were subsequently used in the analysis presented in Figure 4.
