# Supplementary material for: Fusion of a bacterial cysteine desulfurase to redox-sensitive green fluorescent protein produces a highly sensitive cysteine biosensor for monitoring changes in intracellular cysteine
Source: Redox Biol. 2025 Jul 23;85:103785. doi: 10.1016/j.redox.2025.103785 (PMC12319549; doi:10.1016/j.redox.2025.103785)
Supplement: Multimedia component 2 [file mmc2.docx]

**Appendix B. Supplementary Tables**

**Supplementary Table 1. Statistical analysis of OxD values before and after addition of cysteine to *E.* *coli* cells.**

| **Tukey's multiple comparison test** | **Mean Difference** | **95% Confidence Intervals of difference** | **Significant** | **Adjusted P Value** |
| --- | --- | --- | --- | --- |
| WT - CyReB:Before cysteine vs. WT - CyReB:After cysteine | -0.6821 | -0.7589 to -0.6053 | **** | < 0.0001 |
| WT - C334S:Before cysteine vs. WT - C334S:After cysteine | 0.02249 | -0.05433 to 0.09930 | ns | 0.9997 |
| Δ*gshA* - CyReB:Before cysteine vs. Δ*gshA* - CyReB:After cysteine | -0.045 | -0.1218 to 0.03181 | ns | 0.8012 |
| Δ*gshA* - C334S:Before cysteine vs. Δ*gshA* - C334S:After cysteine | -0.1427 | -0.2195 to -0.06586 | **** | < 0.0001 |
| Δ*grxA/B/C* - CyReB:Before cysteine vs. Δ*grxA/B/C* - CyReB:After cysteine | -0.07066 | -0.1475 to 0.006154 | ns | 0.1098 |
| Δ*grxA/B/C* - C334S:Before cysteine vs. Δ*grxA/B/C* - C334S:After cysteine | -0.05045 | -0.1273 to 0.02636 | ns | 0.6365 |
| Δ*trxA/C* - CyReB:Before cysteine vs. Δ*trxA/C* - CyReB:After cysteine | -0.3659 | -0.4427 to -0.2891 | **** | < 0.0001 |
| Δ*trxA/C* - C334S:Before cysteine vs. Δ*trxA/C* - C334S:After cysteine | 0.2905 | 0.2137 to 0.3673 | **** | < 0.0001 |

**Supplementary Table 2. Statistical analysis of OxD values before and after addition of cystine to *E.* *coli* cells.**

| **Tukey's multiple comparison test** | **Mean Difference** | **95% Confidence Intervals of difference** | **Significant** | **Adjusted P Value** |
| --- | --- | --- | --- | --- |
| WT - CyReB:Before cystine vs. WT - CyReB:After cystine | -0.2776 | -0.3563 to -0.1988 | **** | < 0.0001 |
| WT - C334S:Before cystine vs. WT - C334S:After cystine | -0.05123 | -0.1300 to 0.02751 | ns | 0.6519 |
| Δ*gshA* - CyReB:Before cysteine vs. Δ*gshA* - CyReB:After cysteine | -0.05868 | -0.1374 to 0.02006 | ns | 0.4119 |
| Δ*gshA* - C334S:Before cysteine vs. Δ*gshA* - C334S:After cysteine | -0.09063 | -0.1694 to -0.01189 | ** | 0.0088 |
| Δ*grxA/B/C* - CyReB:Before cysteine vs. Δ*grxA/B/C* - CyReB:After cysteine | -0.07005 | -0.1488 to 0.008689 | ns | 0.1438 |
| Δ*grxA/B/C* - C334S:Before cysteine vs. Δ*grxA/B/C* - C334S:After cysteine | -0.1415 | -0.2202 to -0.06275 | **** | < 0.0001 |
| Δ*trxA/C* - CyReB:Before cysteine vs. Δ*trxA/C* - CyReB:After cysteine | -0.2352 | -0.3140 to -0.1565 | **** | < 0.0001 |
| Δ*trxA/C* - C334S:Before cysteine vs. Δ*trxA/C* - C334S:After cysteine | 0.06791 | -0.01083 to 0.1466 | ns | 0.1807 |
